# The association of dietary carbohydrate quality and quantity with obesity among Iranian adolescents: a case-control study

**DOI:** 10.1186/s12887-024-04671-9

**Published:** 2024-03-09

**Authors:** Shabnam Mohebati, Mahboobeh Shakeri, Sara Ranjbar, Mohammad Jalali, Mehran Nouri, Shiva Faghih

**Affiliations:** 1 Department of Community Nutrition, School of Nutrition and Food Sciences, Shiraz University of Medical Sciences, Shiraz, Iran; 2 Endoocrine and Metabolism Research Center, Isfahan University of Medical Sciences, Isfahan, Iran; 3 Department of Nutritional sciences, Texas Tech University, Lubbock, TX USA; 4 Nutrition and Endocrine Research Center, Research Institute for Endocrine Sciences, Shahid Beheshti University of Medical Sciences, Tehran, Iran; 5 Non-Communicable Pediatric Diseases Research Center, Health Research Institute, Babol University of Medical Sciences, Babol, Iran

**Keywords:** Adolescent, Carbohydrate quality, Carbohydrate quantity, Obesity

## Abstract

**Background:**

Adolescent obesity is considered as a major health concern worldwide which is closely linked to the quality of diet. The purpose of the present study was to assess the carbohydrate quality and quantity in relation to odds of overweight and obesity in adolescents.

**Methods:**

This case-control study with a 1:1 ratio was conducted on 406 adolescents (14 to 18 years old) matched for age and gender. Participants were selected by multistage cluster random sampling method from March to October 2019 in Shiraz, Iran. Dietary intakes of the study population were assessed by a validated semi-quantitative food frequency questionnaire. Also anthropometric indices were measured using standard methods and demographic information was recorded via face to face interview. The relation between low carbohydrate diet score (LCDS) and carbohydrate quality index (CQI), and odds of obesity was evaluated by multiple Logistic regression.

**Results:**

After adjusting the role of potential confounders, the participants in the third tertiles of LCDS (OR = 0.443, 95% CI = (0.260 to 0.755)) and CQI (OR = 0.005, 95% CI = (0.001 to 0.025)) had less odds of being overweight and obese compared to the first tertile.

**Conclusion:**

The present study found an inverse relationship between dietary quantity and quality of carbohydrate intake and the odds of overweight and obesity in a sample of Iranian adolescents.

## Introduction

Adolescent obesity, which is closely associated with adulthood obesity, is a health concern in the world [1], and its prevalence was estimated equal to 5.8% in Iranian students [2]. Obesity is linked with higher risk of health issues such as hypertension, and type two diabetes [3], as well as psychological ramification including stigmatization and peer victimization [4].

Various approaches have been suggested to prevent and treat adolescent obesity, including dietary interventions with different ratios of carbohydrates, proteins, and fats [5–7]. Among these patterns, low carbohydrate diets have emerged as a popular approach, which could promote weight loss and improve metabolic outcomes [8, 9]. However, the link among total intake of carbohydrate and obesity risk remains inconclusive. A meta-analysis study found no relationship between higher carbohydrate intake and obesity risk [10], while some others have conflicting results [11, 12]. Moreover, limited research exists regarding the association of low carbohydrate diet score (LCDS) and adolescent obesity [13].

Most studies on carbohydrate intake and obesity have independently assessed various aspects of carbohydrate quality, such as fiber consumption [14], glycemic index (GI) [15], and load (GL) [16], sugar-sweetened beverages [17], and whole grains [18]. But, the relationship between these individual components and obesity has remained controversial. Carbohydrate Quality Index (CQI) is a valuable measure of carbohydrate quality which encompasses multiple individual components, including GI, dietary fiber intake, the proportion of whole to total cereals and the solid to total carbohydrates ratio [19].

The link between CQI with obesity risk and some chronic diseases in adults has been previously evaluated [19–21]. Also, there have been researches on the association between adolescent obesity with dietary pattern and carbohydrate intake quantity [22–24]. However, few studies have been conducted to investigate the connection between CQI and LCDS with obesity in adolescents. Therefore, the purpose of the present study was to assess the relationship between quality and quantity of carbohydrate intake with overweight and obesity in adolescents.

## Materials and methods

### Study design

The current case-control study with a 1:1 ratio (normal weight: overweight or obese) was done on 406 adolescents from March to October 2019, according to the guidelines in the Declaration of Helsinki. The Ethics Committee of Shiraz University of Medical Sciences approved all procedures of the present study (No.IR.SUMS.REC.1398.1310). All participants were informed about the study protocol then written consents were signed.

### Participants

Inclusion criteria were 14–18 years old healthy adolescents. Overweight or obese adolescents (BMI > 85th percentile) were considered as the case group and normal weight adolescents (BMI between 3th and 85th percentile) as the control group. Exclusion criteria were as follows: adolescents who were diagnosed with a disease affecting their nutritional status such as diabetes, cancer, renal failure, liver disease, rheumatic and skeletal disease, or following a dietary regimen that may affect body weight such as, vegetarian, low calorie or high calorie diets.

Participants were selected using multistage cluster random sampling method and matched for age and sex. As school systems are separated based on gender (male schools and female schools) in Iran, we considered 2 stratums for the sampling: educational district and gender. In each of the four educational districts, four high schools were selected using systematic random sampling. Then within each school, classrooms were selected using a simple random sampling method followed by students’ selection using systematic random sampling. The number of students selected in every district was proportionate to the entire population of that district. Also, in the control group, participant’s selection were done from the same classroom which was expected to be in the same social context as the case group.

### Participants’ characteristics

Demographic information including age, sex, school grade, housing ownership, birth order, parents’ education, and family income were recorded via face to face interview. Physical activity was assessed by international physical activity questionnaire (IPAQ) based on metabolic equivalent rate by hours per week (MET/h/W) [25].

### Dietary intakes

Dietary intakes of the study participants were assessed using a validated 147-item semi-quantitative food frequency questionnaire (FFQ) [26]. Frequency of the food items were reported as daily, weekly, monthly and yearly. Then, the frequency was changed to gram by multiplying portion size of food item by frequency with household measurement guidelines for Iranian population. Finally, daily dietary energy and macronutrients intakes were estimated using Nutritionist IV software [27].

### Anthropometric assessment

A digital scale (Omron, China) was used for weight measurement in light clothing. Also, non-elastic tape was used for height assessment. Body mass index (BMI) was calculated as weight (kg) divided by height in meters squared (m^2^).

The NCHS/CDC (2000) cut-off points were used to determine overweight and obesity. Based on BMI-for-sex and –age, below the 85th, between the 85th and 95th, and above the 95th percentile were defined as normal weight, overweight and obese respectively [28]. Also, for the waist circumference (WC) assessment an un-stretchable tape measure was used.

### Exposure measurement

To obtain CQI four criteria were computed: total intake of fiber (g/day), proportion of whole to total cereals (refined and whole cereals), glycemic index (GI), and proportion of solid to total carbohydrate intake (solid and liquid). Fruit juice and sugar-sweetened beverages were summed up to calculate liquid carbohydrates and the rest of carbohydrate content was considered for solid carbohydrates. Each of these four criteria got a score from 1 to 5. Finally, the range of CQI was among 4 to 20. The higher points mean more closely to CQI [29].

LCDS was calculated as follows: participants were allocated in to 11 classes for each components (carbohydrates, plant proteins, refined cereals, monounsaturated and polyunsaturated fatty acids (MUFAs and PUFAs), GL and fibers). In groups of vegetable proteins, MUFAs, PUFAs and fibers, individuals received 10 points for the last stratum and 9 points for the next stratum and so on, the first stratum received 0. However, for carbohydrates, GL and refined grains, the scoring method was reversed (the first stratum received 10 points and the last one received 0). The total points were summed up and the range was from 0 (minimum consumption of protein and fat and maximum consumption of carbohydrate) to 70 (minimum intake of carbohydrate and maximum intake of protein and fat). The higher points mean more closely to LCDS [30, 31].

### Statistical analysis

All statistical analyses were done by Statistical Package for the Social Sciences (SPSS software; ver. 23). Normal distribution of the variables was checked by Kolmogorov-Smirnov test. The values have been shown as mean ± standard deviation (SD) or percent. To compare the variables between control and case groups, independent sample t-test and Chi-square were applied. Also, for comparison the categorical and continuous variables between CQI and LCDS tertiles one way ANOVA was used. The logistic regression in two crude and adjusted models were used to assess the relationship between LCDS and CQI with overweight and obesity. Also, we adjusted the role of potential confounders in the last model.

## Results

### Baseline characteristics

Data 406 adolescents were included to the analysis (203 cases and 203 controls). BMI, WC, GI, dietary intakes of energy, carbohydrate, protein and fats were significantly upper (*P* = 0.001 for all), but, GL was significantly lower in the case group in comparison to the control group (*P* = 0.001) (Table 1).

**Table 1:** Characteristics of the study participants

Variables	Case (*N* = 203)	Control (*N* = 203)	P-value
Age (year)	16.28 ± 0.64	16.25 ± 0.68	0.708
BMI (kg/m^2^)	29.08 ± 3.54	21.09 ± 1.85	**0.001**
WC (cm)	92.78 ± 10.17	75.28 ± 5.11	**0.001**
Sex, boy (%)	51.7	51.7	1.000
Physical activity (%)			0.135
Low Moderate High	14.3 54.7 31.0	10.3 49.8 39.9	
Income (%)			0.154
Low Moderate High	55.2 40.4 4.4	45.8 47.8 6.4	
Energy (Kcal/day)	2863.49 ± 212.43	2554.28 ± 141.69	**0.001**
CHO (g/day)	396.94 ± 29.86	370.19 ± 34.42	**0.001**
Protein (g/day)	101.33 ± 7.06	90.13 ± 5.89	**0.001**
Fat (g/day)	101.06 ± 10.61	83.69 ± 4.10	**0.001**
GI	63.20 ± 1.10	61.11 ± 1.57	**0.001**
GL	85.47 ± 3.17	87.20 ± 6.39	**0.001**

BMI: body mass index; WC: waist circumference; CHO: carbohydrate; GI: glycemic index; GL: glycemic load

Values are mean ± standard deviation or percent

P-value less than 0.05 was considered significant

Independent Sample t-Test and chi-square have been used

Significant values are shown in bold

As shown in Table 2, participants with higher LCDS had greater intake of dietary fibers (g/day) and MUFAs (% energy). On the other hand, BMI, WC, GI and GL scores, energy, carbohydrate, refined grain intakes were significantly lower in the last tertile of LCDS in comparison to the first one.

**Table 2:** Characteristics of the study participants based on LCDS tertiles

Variables	LCDS			P-value
	T_1_ (*N* = 127)	T_2_ (*N* = 145)	T_3_ (*N* = 134)	
Age (year)	16.23 ± 0.73	16.23 ± 0.68	16.33 ± 0.54	0.357
BMI (kg/m^2^)	25.64 ± 4.52	25.48 ± 5.24	24.13 ± 4.74	**0.022**
WC (cm)	86.14 ± 10.54	85.02 ± 12.52	80.96 ± 11.86	**0.001**
Sex, boy (%)	80.3	60.7	51.7	**< 0.001**
Physical activity (%)				**< 0.001**
Low Moderate High	5.5 40.2 54.3	14.5 50.3 35.2	16.4 65.7 17.9	
Family Income (%)				0.113
Low Moderate High	56.7 37.8 5.5	51.7 41.4 6.9	43.3 53.0 3.7	
Energy (Kcal)	2815.27 ± 140.61	2749.97 ± 231.35	2563.59 ± 247.78	**< 0.001**
CHO (% energy)	58.43 ± 2.08	56.69 ± 2.08	54.94 ± 1.63	**< 0.001**
Fibers (g/day)	4.96 ± 0.68	5.01 ± 0.65	5.42 ± 0.76	**< 0.001**
MUFA (% energy)	9.48 ± 0.69	9.98 ± 0.81	10.55 ± 0.67	**< 0.001**
Fatty acid N_3_ / N_6_ ratio	0.045 ± 0.023	0.044 ± 0.027	0.047 ± 0.023	0.697
Refined cereals (g/day)	419.20 ± 15.73	393.40 ± 22.22	355.34 ± 33.12	**< 0.001**
GI	63.00 ± 1.40	62.21 ± 1.14	61.29 ± 2.05	**< 0.001**
GL	90.67 ± 3.65	86.93 ± 3.46	81.58 ± 3.59	**< 0.001**
Plant proteins (% energy)	2.88 ± 0.27	2.90 ± 0.33	2.95 ± 0.26	0.122

LCDS: low carbohydrate diet score; BMI: body mass index; WC: waist circumference; CHO: carbohydrate; GI: glycemic index; GL: glycemic load

Values are mean ± standard deviation or percent

P-value less than 0.05 was considered significant

One-way ANOVA and chi-square have been used

Significant values are shown in bold

Regarding CQI, BMI, WC, energy intake, dietary proteins, fats, liquid CHO and GI were lower in the third tertile comparing the first one (*P* < 0.001). However, dietary whole cereals, total cereals and solid CHO were higher in the participants in the last tertile (Table 3).

**Table 3:** Characteristics of the study participants based on CQI tertiles

	CQI			P-value
Variables	**T**_**1**_ (*N* = 128)	**T**_**2**_ (*N* = 145)	**T**_**3**_ (*N* = 133)	
Age (year)	16.21 ± 0.68	16.32 ± 0.59	16.25 ± 0.70	0.409
BMI (kg/m^2^)	29.08 ± 4.47	25.03 ± 4.26	21.29 ± 2.12	**< 0.001**
WC (cm)	94.09 ± 11.87	82.84 ± 9.70	75.65 ± 4.99	**< 0.001**
Sex, boy (%)	71.9	21.4	65.4	**< 0.001**
Physical activity (%)				**< 0.001**
Low Moderate High	10.9 47.7 41.4	14.5 64.8 20.7	11.3 42.9 45.9	
Income (%)				**0.032**
Low Moderate High	60.9 35.2 3.9	46.9 49.0 4.1	44.4 47.4 8.2	
Energy (kcal)	2915.57 ± 225.16	2630.36 ± 207.84	2595.57 ± 127.53	**< 0.001**
Protein (g/day)	102.48 ± 7.80	92.90 ± 8.22	92.32 ± 5.37	**< 0.001**
Fat (g/day)	102.36 ± 11.54	92.43 ± 9.39	82.71 ± 3.90	**< 0.001**
Whole cereals (g/day)	16.81 ± 3.63	21.95 ± 4.09	28.06 ± 7.52	**< 0.001**
Total cereals (g/day)	93.56 ± 4.83	92.45 ± 7.43	101.51 ± 9.24	**< 0.001**
Liquid CHO (g/day)	15.01 ± 2.29	11.88 ± 1.82	10.89 ± 1.09	**< 0.001**
Solid CHO (g/day)	135.72 ± 3.63	136.35 ± 5.68	146.42 ± 6.93	**< 0.001**
GI	63.38 ± 1.16	61.87 ± 1.75	61.27 ± 1.42	**< 0.001**

CQI: carbohydrate quality index; BMI: body mass index; WC: waist circumference; CHO: carbohydrate; GI: glycemic index

Values are mean ± standard deviation or percent

P-value less than 0.05 was considered significant

One-way ANOVA and chi-square have been used

Significant values are shown in bold

After adjusting the role of potential confounders in the last model, a reverse association were seen between LCDS (OR = 0.443, 95% CI = (0.260 to 0.755)) and CQI (OR = 0.005, 95% CI = (0.001 to 0.025)) with overweight and obesity risk in the last tertile compared to the first one (Table 4).

**Table 4:** Relation of overweight and obesity with LCDS and CQI in the study population

Variables	T_1_	T_2_	T_3_	P-value
		OR (95% CI)	OR (95% CI)	
**LCDS**				
Crude model	Ref.	0.975 (0.604, 1.573)	**0.550 (0.336, 0.898)**	**0.016**
Adjusted model^&^	Ref.	0.869 (0.529, 1.427)	**0.443 (0.260, 0.755)**	**0.003**
**CQI**				
Crude model	Ref.	**0.075 (0.034, 0.166)**	**0.003 (0.001, 0.009)**	**< 0.001**
Adjusted model^&^	Ref.	**0.063 (0.028, 0.144)**	**0.003 (0.001, 0.009)**	**< 0.001**
Adjusted model^*^	Ref.	**0.179 (0.052, 0.615)**	**0.005 (0.001, 0.025)**	**< 0.001**

LCDS: low carbohydrate diet score, CQI: carbohydrate quality index

^&^Adjusted for: physical activity (low, moderate or high) and income (low, moderate or high)

^*^Adjusted for: physical activity (low, moderate or high), income (low, moderate or high) and energy intake (kcal/day)

Values are odd ratio and 95% CI.

Obtained by logistic regression

Significant values are shown in bold

## Discussion

The current study assessed the link between LCDS and CQI with overweight and obesity risk in adolescents. The findings showed significant relationships between CQI as well as LCDS with obesity both before and after adjustment for potential confounders.

Our findings indicated that as CQI improves both general and abdominal obesity decreases. These results are consistent with some previous reports [19, 20, 32] while few studies did not find similar associations [33]. Findings of a cross-sectional research on the association of metabolic syndrome in adults with habitual and meal specific CQI showed no significant relationship between CQI and weight (27). Nonetheless, two other cross-sectional studies on 277 women in Ghana [19] and 12,027 adult participants, consisting both men and women, in South Korea [32], along with a cohort study with a follow up of 7.9 years involving 8741 adults found inverse association of CQI with BMI and WC [20]. Individual components of CQI, such as higher intake of fibers, whole grains [34, 35], and solid carbohydrates also low intake of liquid carbohydrates [36], could help lower the risk of obesity and overweight.

Additionally, this study revealed a link between LCDS and general and abdominal obesity. In this regard, other studies have reported conflicting findings [10, 11, 37, 38]. Opposite to our results, a meta-analysis of 22 articles showed no significant association between high carbohydrate diet and obesity. However, the review did mention that non-standardized dietary records, heterogeneity across studies, and lack of quantification of different carbohydrate classes in some studies were of important limitations [10]. In another study involving adult women, no significant relationship was found between weight and LCDS. This lack of association might be due to the fact that the most women had normal BMI and waist circumferences. Furthermore, there were no significant differences in energy intake among the tertiles [39].

In support of our results a cross-sectional study on women in Sri Lanka showed positive correlation between high carbohydrate intake and WC [37]. A study in Korea demonstrated an independent association of white rice and sweets pattern with obesity while low carbohydrate patterns had not significant association [40].

The relationship between CQI and obesity can be explained through promoting satiety, slowing nutrients absorption, influencing gut hormones secretions and supporting gut microbiome. Diets with a high CQI also have high fiber contents, which help reduce weight. Fibers can be helpful in weight reduction by improving satiety, suppressing appetite, reducing food intake, and enhancing insulin sensitivity [41, 42]. Whole grains are also another component of CQI whose consumption prevents weight gain by prolonging intestinal transit time, increasing energy expenditure, and improving satiety and insulin sensitivity [43]. Another component of CQI connected to obesity is the solid to total carbohydrate ratio. Liquid carbohydrates are often rich in sugar and have a high GI. These beverages could raise the risk of obesity through a variety of mechanisms, including impaired glucose homeostasis, increased appetite and insulin resistance [36, 44].

A plausible explanation supporting the relation between LCDS and weight is that low-carbohydrate diets may lower calorie consumption physiologically by stimulating energy expenditure via gluconeogenesis. By reduction of insulin concentration, it leads to usage of fat storage and consequently weight loss [45]. Further, low carbohydrate diets stimulate weight reduction by reducing food intake due to decreased levels of ghrelin and leptin hormones [46, 47]. Increased production of ketone bodies as induces by LCD ghrelin levels tend to decrease [48]. Low carbohydrate diets can enhance leptin sensitivity which leads to reduced appetite and increased satiety [49]. Besides, low GL diets improve satiety by decreasing voluntary food intake and consequently total calorie consumption [50, 51].

This study provides a valuable contribution to the field, however several limitations must be noted. By narrowing down the study to adolescents’ age and considering both quantity and quality of carbohydrate, the research provide a more targeted and contextualized findings. A limitation for our study was administering the questionnaire retroactively. This may limit information recall and lead to under or over reporting of intakes. Furthermore, despite the fact that certain possible confounders were adjusted when analyzing, the impacts of eating habits, psychological and sociocultural status, and genetic variants were not considered in this research.

## Conclusion

The present study found a reverse relationship between CQI and LDCS with overweigh and obesity risk in adolescents. The findings give critical information that may benefit planning in the prevention and management of obesity in children and adolescents. Larger studies with more participants from various regions and nations, as well as well-designed clinical trials, should be done to better clarify this association.

## Data Availability

**and methods**: To protect study participant privacy, data cannot be shared openly, but data are available through a reasonable request from the corresponding author.
